# Optimum conductive fabric sensor sites for evaluating the status of knee joint movements using bio-impedance

**DOI:** 10.1186/1475-925X-10-48

**Published:** 2011-06-06

**Authors:** Byung Woo Lee, Chungkeun Lee, Jinkwon Kim, Myoungho Lee

**Affiliations:** 1 Medical Electronics and Information Lab., Department of Electrical & Electronic Engineering, Yonsei University

## Abstract

**Background:**

There have been many studies that utilize the bio-impedance measurement method to analyze the movements of the upper and lower limbs. A fixed electrical current flows into the limbs through four standard disposable electrodes in this method. The current flows in the muscles and blood vessels, which have relatively low resistivity levels in the human body. This method is used to measure bio-impedance changes following volume changes of muscles and blood vessels around a knee joint. The result of the bio-impedance changes is used to evaluate the movements. However, the method using the standard disposable electrodes has a restriction related to its low bio-impedance changes: the standard disposable electrodes are only able to measure bio-impedance from a limited part of a muscle. Moreover, it is impossible to use continuously, as the electrodes are designed to be disposable. This paper describes a conductive fabric sensor (CFS) using a bio-impedance measurement method and determines the optimum configuration of the sensor for estimating knee joint movements.

**Methods:**

The upper side of subjects' lower limbs was divided into two areas and the lower side of subjects' lower limbs was divided into three areas. The spots were matched and 6 pairs were selected. Subjects were composed of 15 males (age: 30.7 ± 5.3, weight: 69.8 ± 4.2 kg, and height: 173.5 ± 2.8 cm) with no known problems with their knee joints. Bio-impedance changes according to knee joint flexion/extension assessments were calculated and compared with bio-impedance changes by an ankle joint flexion/extension test (SNR I) and a hip joint flexion/extension test (SNR II).

**Results:**

The bio-impedance changes of the knee joint flexion/extension assessment were 35.4 ± 20.0 Ω on the (1, 5) pair. SNR I was 3.8 ± 8.4 and SNR II was 6.6 ± 7.9 on the (1, 5) pair.

**Conclusions:**

The optimum conductive fabric sensor configuration for evaluating knee joint movements were represented by the (1, 5) pair.

## Background

As the number of people who have problems with their joints increases, the need for joint rehabilitation cures has gradually risen. The past status and future status of joint rehabilitation are always estimated in the curing phase of joint rehabilitation. In particular, the status of knee joint movement as regards rehabilitation is evaluated using goniometers to measure knee joint angles, active and passive marker systems to detect motion, and electromyography (EMG) to measure muscle fatigue around knee joints [1].

There have been many studies that utilize the bio-impedance measurement method to analyze the movements of the upper and lower limbs [2] and [3]. A fixed electrical current flows into the limbs through four standard disposable electrodes in this method. The current flows in the muscles and blood vessels, which have relatively low resistivity levels in the human body. This method is used to measure bio-impedance changes following volume changes of muscles and blood vessels around a knee joint. The result of the bio-impedance changes is used to evaluate the movements [4] and [5]. However, the method using the standard disposable electrodes has a restriction related to its low bio-impedance changes: the standard disposable electrodes are only able to measure bio-impedance from a limited part of a muscle. Moreover, it is impossible to use continuously, as the electrodes are designed to be disposable.

This paper described a strip-type conductive fabric sensor (CFS) which includes much more muscle areas compared to the standard disposable electrodes using the bio-impedance measurement method. Bio-impedance changes caused by knee joint flexion/extension using the strip-type CFS were calculated and compared with bio-impedance changes caused by ankle joint flexion/extension (SNR I) and hip joint flexion/extension (SNR II). As the measured results were compared, we could determine the optimum configuration of the strip-type CFS to estimate knee joint movements.

## Methods

### 1. The Strip-type Conductive Fabric Sensor (CFS)

The equation of bio-impedance is given as follows, from Nyboer (1970) and Swanson (1976):(1)

In equation (1), Z is the bio-impedance of the cylindrical limbs, L is the distance among the electrodes, A is the area of sensing, and ρ is the characteristic resistivity of the muscles and blood vessels.

Equations (2) and (3) show the bio-impedance of the muscles (Z_m_) and the blood vessels (Z_b_) on the cylindrical limbs [6] and [7].(2)(3)

Furthermore, if L is fixed, the more the areas of sensing on the muscles (ΔA_m_) and the blood vessels (ΔA_b_) increase, the more the volume variations of the muscles (ΔV_m_) and the blood vessels (ΔV_b_) can be measured from equations (4) and (5).(4)(5)

When moving a knee joint, the total volume variations (ΔV) of the increases in the muscles (ΔV_m_) and blood vessels (ΔV_b_) are determined as follows:(6)

This shows, in conjunction with equation (6), that the sensing areas of limbs should be expanded when measuring the volume variations of increases in muscles and blood vessels.

**Figure 1:**
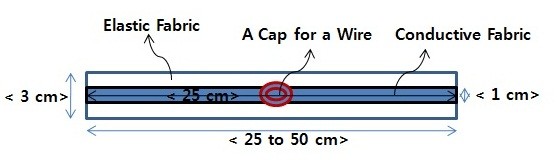
**The Manufactured Strip-type Conductive Fabric Sensor to Measure Bio-impedance from Cylindrical Limbs**.

Therefore, the strip-type conductive fabric sensor (CFS) measured 25 cm by 1 cm based on an expansion of the sensing areas of the limbs, as shown in Figure 1. In addition, the external part of the sensor consisted of contractible non-conductive fabric, as the volume of each subject's muscles differed. The CFS has an adjustable range of 25 cm by 3 cm to 50 cm by 3 cm.

The proposed strip-type CFS, W-290-PCN, was produced by Ajin electrons(Busan, Korea). The CFS was made of polyester and Ni-Cu-Ni and was produced by electro less plating method in order to be stronger the plating adhesive property. The surface resistance of the CFS has a range from 0.005 Ω/sq to 50 Ω/sq.

### 2. System and Sensor Configuration

MP150, EBI100C, and Lead130 (BIOPAC Systems) devices were used to determine optimum CFS configuration to evaluate knee joint movements. MP150 was used as an A/D converter and a communicator with a computer, EBI100C was used as a device to flow a fixed electrical current into a human body and an amplifier to magnify the bio-impedance, and Lead130 was used as a lead to measure bio-impedance. The excitation frequency on the CFS must be in the range of 20 kHz to 100 kHz, and the electric current can flow into a human body up to 4 ㎃ to detect the bio-impedance [4] and [5]. The devices can send out the excitation frequencies of 25 kHz, 50 kHz, and 100 kHz and a fixed electrical current of 100 ㎂ in a human body. Therefore, these devices are suitable for measuring bio-impedance changes.

A tilt sensor (SCA61T-FA1H1G, VTI Technologies) with a range of ± 90° was used to measure the angle changes of a knee joint and verify the movements of the knee joint. The sensitivity of the tilt sensor resulting from angle changes was 35 mV/° or 2 V/g. The offset voltage of the tilt sensor was 2.5 V.

The upper side (from the hip joint to the knee joint) of the subjects' lower limbs was divided into two areas and the lower side (from the knee joint to the ankle joint) of the subjects' lower limbs was divided into three areas. The CFS was then attached to two positions denoted as 1 of 3 and 2 of 3 from the hip joint on the upper side and to three positions denoted as 1 of 4, 2 of 4, and 3 of 4 from the knee joint on the lower side in turns. These positions are based on optimum positions for measuring the bio-impedance of the lower limbs using the standard disposable electrodes [2] and [3].

**Figure 2:**
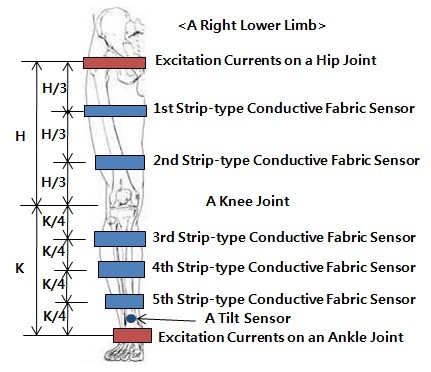
**The Positions of the Strip-type Conductive Fabric Sensor on Lower Limbs to Determine Optimum CFS Configuration to Evaluate Knee Joint Movements**.

Figure 2 shows the sites at which the CFS was attached. Each spot was matched to another spot, resulting in 6 pairs selected. In addition, a tilt sensor was attached to the muscles above the ankle joint to measure the angle changes of the knee joint and verify the movements of the knee joint.

### 3. Experimental Methods

The CFS was excited at 50 kHz and 100 ㎂ which are the optimum frequency and the electrical current of the MP150 and EBI100C (BIOPAC Systems) devices. The bio-impedance and tilt signal were measured from the CFS through the knee, ankle, and hip joints flexions/extensions for every 1 pair for 60 seconds. At this time, the knee, ankle, and hip joints flexions/extensions were matched by the rising/falling of the trigger signals generated in a generator device. The experiments were conducted using six pairs in total. Subjects were composed of 15 males (age: 30.7 ± 5.3, weight: 69.8 ± 4.2 kg, and height: 173.5 ± 2.8 cm) who did not report any problems with their knee joints. The study conformed to the Declaration of Helsinki, and approved by the local research ethics committee. All subjects gave written informed consent.

## Results

**Figure 3:**
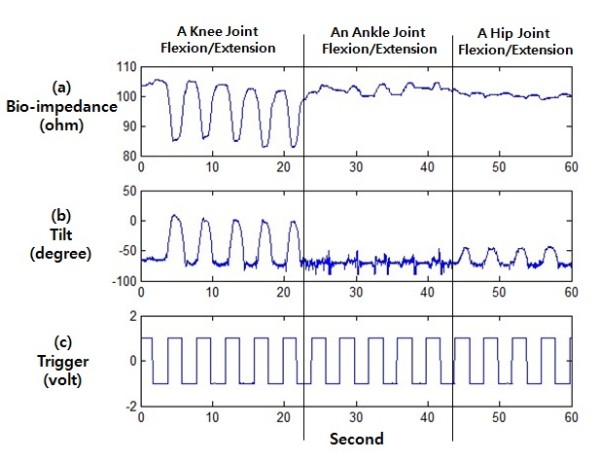
**The Bio-impedance and Knee Angle Changes by the Knee, Ankle, and Hip Joints Flexions/Extensions Followed by Trigger Signal for 60 Seconds on the Sensor Pair (1, 5) of a Subject**.

Figure 3 represents the bio-impedance and knee joint angle changes matched by the rising/falling of the trigger signals during knee, ankle, and hip joints flexions/extensions on the sensor pair (1, 5) of a subject.

Figure 3(a) shows bio-impedance changes following the knee, ankle, and hip joints flexions/extensions. Tilt signals attached to a part of the muscles above the ankle joint to measure the angle changes of the knee joint and verify the movements of the knee joint are represented in Figure 3(b). Figure 3(c) shows the trigger signals, which have a period of 15 times for every 60 seconds, signifying 250 mHz from a generator device. Subjects conducted knee, ankle, and hip joints flexions upon rising trigger signals, and knee, ankle, and hip joints extensions upon falling trigger signals.

The strip-type CFS was attached onto the following 6 pairs: (1, 3), (1, 4), (1, 5), (2, 3), (2, 4), and (2, 5) by turns per subject. Bio-impedance changes by a knee joint flexion/extension were calculated and compared with bio-impedance changes caused by an ankle joint flexion/extension (SNR I) and a hip joint flexion/extension (SNR II).

Table 1 represents the average values and standard deviations of the bio-impedance changes, SNR I results, and SNR II results from the 15 male subjects.

**Table 1:** The Rank of Sensor Pairs by Average Bio-impedance Changes, SNR I and SNR II Measured from 15 Male Subjects

Sensor Pairs	Bio-impedance Changes (Ω)	SNR I	SNR II	Scores	Ranks
(1, 5)	35.4 ± 20.0 (3)	3.8 ± 8.4 (1)	6.6 ± 7.9 (3)	7	1
(2, 5)	23.2 ± 12.2 (5)	2.2 ± 7.3 (2)	7.4 ± 10.9 (2)	9	2
(2, 3)	81.5 ± 135.4 (1)	0.8 ± 8.7 (4)	2.2 ± 7.4 (5)	10	3
(1, 4)	25.8 ± 24.8 (4)	0.8 ± 7.1 (3)	4.3 ± 9.6 (4)	11	4
(2, 4)	18.1 ± 11.5 (6)	0.5 ± 3.6 (5)	9.2 ± 8.6 (1)	12	5
(1, 3)	54.9 ± 107.3 (2)	-0.5 ± 11.2 (6)	2.2 ± 11.8 (6)	14	6

The highest average value of the bio-impedance changes scored 1 point and the lowest average value of the bio-impedance changes scored 6 points among the 6 pairs. In addition, the highest value of SNR I scored 1 point and the lowest value of SNR I scored 6 points among the 6 pairs. SNR II was also calculated in the same manner. All scored values for the 6 pairs were then summed up. Therefore, the lower the score of the sum was, the better the result of the knee joint movements.

As a result, the score of the sensor pair (1, 5) was 7 points, representing the lowest value of the sum scores. Thus, this sensor pair (1, 5) was verified as the first optimum CFS configuration of the 6 pairs. The second optimum CFS configuration of the 6 pairs was the (2, 5) pair, which scored 9 points.

The standard deviations of the bio-impedance changes had comparatively large values, as shown in Table 1. Although the bio-impedance was measured by the CFS at the same sites when moving the knee joint, the volume changes of each subject's muscle used for moving the knee joint can differ. Consequently, a difference in each subject's bio-impedance change can exist at the same position.

**Figure 4:**
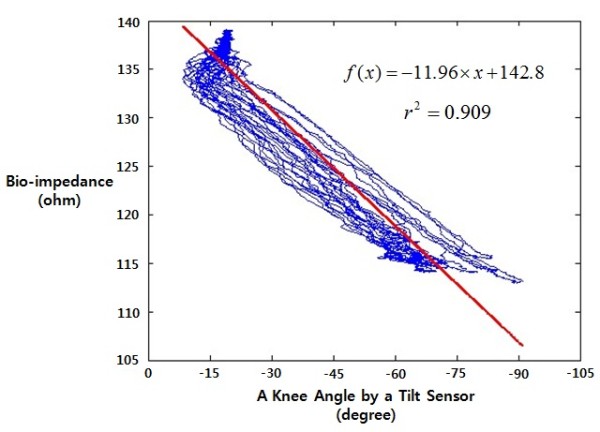
**The Correlation between the Bio-impedance Changes and the Knee Angle Changes on the Optimum Site (1, 5) using the CFS as Moving the Knee Joint**.

Figure 4 shows the correlation between the bio-impedance changes and the knee joint angles for the optimum pair (1, 5) according to the CFS when moving the knee joint. The rows represent the knee joint angles measured from a tilt sensor and the columns refer to the bio-impedance measured from the CFS. R^2^, the result of the linear regression analysis, is 0.909. This indicates that the relationship between the bio-impedance changes and the knee joint angles for the optimum pair (1, 5) according to the CFS when moving the knee joint is statistically significant.

## Discussion

This paper describes a conductive fabric sensor (CFS) using a bio-impedance measurement method and determines the optimum configuration of the sensor to estimate the knee joint movement.

The upper sides of subjects' lower limbs were divided into two areas, and the lower sides of subjects' lower limbs were divided into three areas. The individual spots in each set were matched, and 6 pairs were selected.

The bio-impedance changes caused by a knee joint flexion/extension were calculated and compared with the bio-impedance changes caused by an ankle joint flexion/extension (SNR I) and a hip joint flexion/extension (SNR II).

As a result, the optimum conductive fabric sensor configuration for evaluating knee joint movements was determined to be the (1, 5) pair. The (1, 5) pair ranked third in bio-impedance changes, first in SNR I, and third in SNR II.

This indicates that the resulting pair is appropriate given the equation of Nyboer (1970) and Swanson (1976). Although each subject used different muscles for their knee joint movements, it can be verified that bio-impedance changes increase comparatively when increasing the distance between the CFS on the cylindrical limbs.

The second optimum configuration was the (2, 5) pair, ranked as fifth in bio-impedance changes and second in both SNR I and SNR II. We found the optimum site of the muscle which reflects relatively well knee joint movements through a SNR analysis.

The SNR I/II of (1, 5) and (2, 5) pairs hold comparatively higher ranks than those of other pairs. Consequently, these positions are better sites for knee joint movements.

On the other hand, the (1, 3) pair for which the distance between the CFS was relatively short was ranked second in bio-impedance changes and sixth in both SNR I and SNR II. Although the (2, 4) pair ranked first in SNR I, this pair ranked sixth in bio-impedance and fifth in SNR I.

This implies that these pairs are not good sites for knee joint movements.

As a result, the distance of the CFS was found to be a meaningful factor for determining the optimum configuration. Moreover, though the sites of an individual's muscles as used for the knee joint movements are different from those of another individual, the optimum sites could be determined by the SNR analysis.

Hence, the bio-impedance measurement method using a CFS is not only less expensive than existing methods involving goniometers, active and passive marker systems, and electromyography (EMG) for evaluating knee joint movements, but they are also more convenient. In addition, this method does not require a technically trained operator.

The bio-impedance changes measured by a CFS are relatively large compared to those measured by the standard disposable electrodes. In addition, the CFS is not disposable and can be used continuously for estimating knee joint movements.

In conclusion, the bio-impedance measurement method using a CFS can be widely applied for evaluating knee joint movements based on the optimum configuration presented in this paper.

## Conclusions

Many people have a great interest in exercise as an effort to maintain good health. However, they often experience an injury to their knee joints should they exercise too heavily. Hence, the people will have to rehabilitate their knee joints. Besides, in this case, the status of rehabilitation must be estimated by assessing methods. As one of many methods to evaluate knee joint rehabilitation, bio-impedance measurement method using wearable pants which are made of CFS will be used sensitively and effectively to value the status of knee joints by the optimum CFS configuration presented on this paper. The existing problems which goniometer, active and passive marker system, and EMG have can be solved through bio-impedance measurement method. This method has many advantages, including lower expenses, no restrictions related to location, and no extra signal processes required. In addition, it will be possible to measure the bio-impedance and to evaluate knee joint movements by simply wearing the pants without extra electrodes, sensors, or devices. Therefore, the CFS will be very suitable and useful to apply to various wearable-type technologies, applications, and industries in the near future. As further study, the optimum excitation frequency of the CFS for evaluating the status of knee joint movements using bio-impedance will be researched.

## Competing interests

The authors declare that they have no competing interests.

## Authors' contributions

BWL conceived the study, implemented a conductive fabric sensor, and drafted the manuscript. CKL and JKK participated in the design and coordination of the study and helped analysis and interpretation of the results. MHL finally reviewed the manuscript as corresponding author. Lastly, BWL, CKL, JKK, and MHL have read and approved the final manuscript.
